# Machine learning in the prediction and detection of new-onset atrial fibrillation in ICU: a systematic review

**DOI:** 10.1007/s00540-024-03316-6

**Published:** 2024-04-09

**Authors:** Krzysztof Glaser, Luca Marino, Janos Domonkos Stubnya, Federico Bilotta

**Affiliations:** 1 Department of Anaesthesiology, Critical Care and Pain Medicine, Policlinico Umberto I,, Sapienza University of Rome, 00185 Rome, Italy; 2 Department of Mechanical and Aerospace Engineering, Policlinico Umberto I, Sapienza University of Rome, 00185 Rome, Italy; 3 Semmelweis University, Ulloi ut 26, Budapest, U1085 Hungary

**Keywords:** Machine learning, Atrial fibrillation, New-onset atrial fibrillation, Intensive care unit, Systematic review, Artificial intelligence

## Abstract

Atrial fibrillation (AF) stands as the predominant arrhythmia observed in ICU patients. Nevertheless, the absence of a swift and precise method for prediction and detection poses a challenge. This study aims to provide a comprehensive literature review on the application of machine learning (ML) algorithms for predicting and detecting new-onset atrial fibrillation (NOAF) in ICU-treated patients. Following the PRISMA recommendations, this systematic review outlines ML models employed in the prediction and detection of NOAF in ICU patients and compares the ML-based approach with clinical-based methods. Inclusion criteria comprised randomized controlled trials (RCTs), observational studies, cohort studies, and case–control studies. A total of five articles published between November 2020 and April 2023 were identified and reviewed to extract the algorithms and performance metrics. Reviewed studies sourced 108,724 ICU admission records form databases, e.g., MIMIC. Eight prediction and detection methods were examined. Notably, CatBoost exhibited superior performance in NOAF prediction, while the support vector machine excelled in NOAF detection. Machine learning algorithms emerge as promising tools for predicting and detecting NOAF in ICU patients. The incorporation of these algorithms in clinical practice has the potential to enhance decision-making and the overall management of NOAF in ICU settings.

**Supplementary Information:**

The online version contains supplementary material available at 10.1007/s00540-024-03316-6.

## Introduction

Atrial fibrillation (AF) is the most prevalent arrhythmia in the ICU patients [1], with new-onset atrial fibrillation (NOAF) developing in one of six patients admitted to the ICU [2]. Prediction and early detection NOAF are imperative in ICU settings, given its association with life-threatening complications and prolonged hospital length of stay [1]. Specific triggers in ICU patients, such as structural and electrical remodeling related to infection and inflammation, along with arrhythmogenic triggers like continuous catecholamine infusion, can precipitate NOAF [1]. Clinical suspicion and detection of NOAF often relay on continuous ECG monitoring and analysis of 12-leads ECG analysis [2]. Severe acute complications encompass hemodynamic compromise, peripheral organs embolism, ischemia (brain, kidney, etc.), and death [3–5]. The occurrence of NOAF is linked to a more challenging clinical course, including extended length of stay increased mortality and the development of ICU-acquired weakness in survivors [5–7]. Several models have been proposed to predict and detect NOAF in ICU patients, with the current gold standard being the “post-operative atrial fibrillation (POAF) score’’. However, its clinical utility is constrained by its poor accuracy [8–12].

Machine learning (ML), a subset of artificial intelligence (AI), encompasses algorithms employing statistical and optimization methods to learn from prior data experiences. Its primary goal is to detect or predict valuable outcomes within extensive databases [13–15]. The significant ML development is related to the possible replication of human intelligence in machines programmed to duplicate important cognitive processes such as learning, problem-solving, and decision-making to analyze and process large amounts of data, focused on extracting useful information without being explicitly programmed [16]. ML capabilities extend to the development of procedures that autonomously learn from prior experiences, enhancing knowledge in specific domains. ML-based algorithms represent emerging and promising techniques for early AF detection [17]. These algorithms exhibit the capacity to identify patterns, make predictions, and propose actions [18]. Prediction and detection of NOAF are critical in ICU patients holding the potential to substantially contribute to the complicated clinical courses [1].

Despite the expanding body of evidence regarding the clinical applications of ML in risk stratification and early diagnosis of NOAF in ICU patients, there is a noticeable absence of dedicated reports on the role of ML in this specific context. The primary objective of this systematic review (SR) is to elucidate the existing evidence concerning the role of ML in both predicting and detecting NOAF in ICU patients.

## Materials and methods

### Protocol and registration

This SR was conducted based on the recommendations of the Preferred Reporting and Items for Systematic Reviews and Meta-Analyses (PRISMA) and was recorded in the PROSPERO registry for SR (N. CRD42023397136, Feb 17, 2023).

### Search strategy

A literature search was conducted through PubMed, Embase, Scopus, and Medline databases, covering publications available until May 31, 2023. The search strategy employed the following terms: (atrial fibrillation OR new-onset atrial fibrillation) AND (artificial intelligence OR machine learning) AND (intensive care unit OR ICU).

Inclusion criteria comprised randomized controlled trials (RCTs), observational studies, cohort studies, and case–control studies. Full-text articles published in English, focusing on adult patients (age > 18 years old) treated in the ICU and utilizing ML for predicting the clinical outcome of AF, both pre-existing and new onset, were included. Exclusion criteria encompassed case reports, comments, letters to the editor, editorials, study protocols, and replies. Studies involving pediatric patients and those not published in English were also excluded.

The primary outcomes sought were the ML-based prediction and/or detection of NOAF in patients admitted to the ICU. The assessment focused on evaluating the accuracy, sensitivity, and specificity of the machine learning algorithms in predicting and detecting the occurrence of NOAF.

### Risk of bias assessment in individual studies

The assessment of bias risk encompassed an examination of five key parameters: a sufficiently sized cohort, appropriate cross-validation, an external validation set, blinding of participants and personnel, and handling of incomplete outcome data (Table 1). For data extraction, a predefined form was utilized, capturing essential information such as study type, sample size, patient characteristics, intervention/exposure details, comparator details, outcome measures, effect measures, follow-up time, funding source, and conflicts of interest.

**Table 1:**
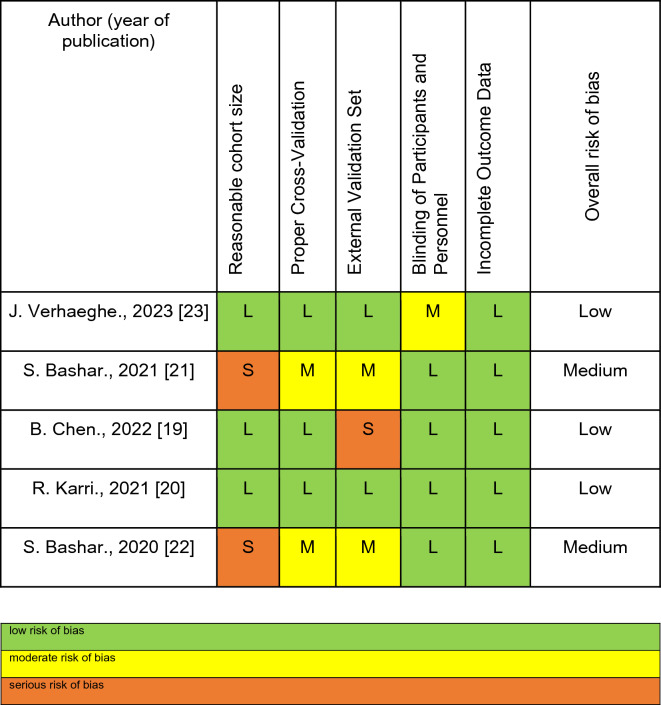
Risk of bias evaluation panel of included studies

Table reports risk of bias evaluation panel

## Results

### Study selection and description

**Fig. 1:**
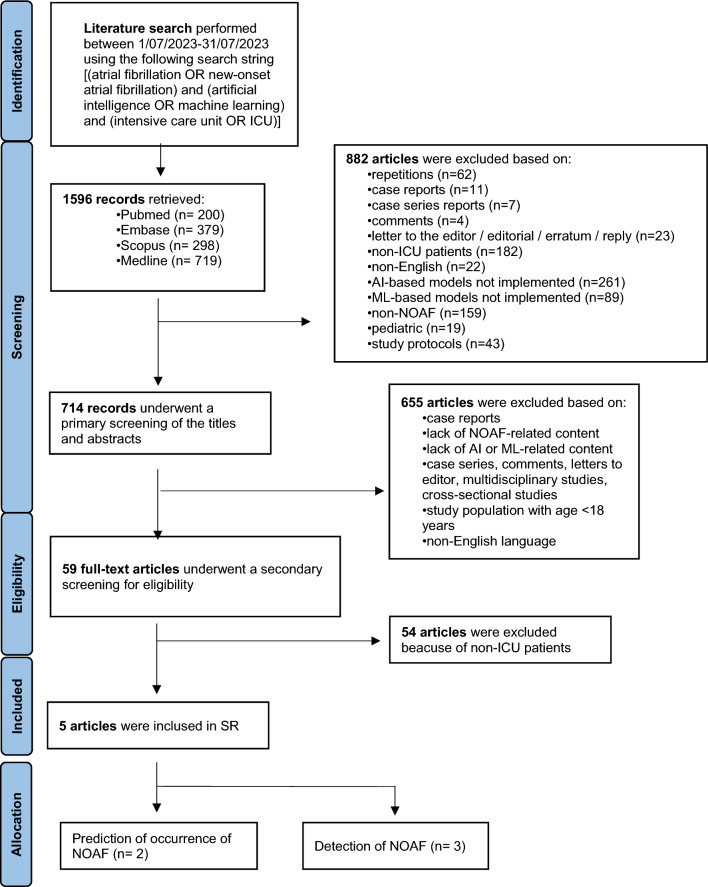
PRISMA diagram of the literature search [30]. Exclusion process flowchart. *AI* artificial intelligence, *ML* machine learning, *NOAF* new-onset atrial fibrillation, *SR* systematic review

Literature search was conducted in July 2023, resulting in the retrieval of 1596 records encompassing risk factors, treatment, prophylaxis, monitoring, and outcomes associated with AF. Among these, 714 articles (44, 7%) were identified as potentially relevant for investigating the role of ML in the prediction and detection of NOAF. Two expert reviewers independently screened the papers, resulting in the selection of 59 articles out of the 714 selected. A subsequent secondary screening focused on excluding studies involving non-ICU patients. Any disparities between the reviewers were resolved through discussion and consensus. Ultimately five studies, each aligning with at least one of the designated outcomes, were deemed eligible for inclusion in the present SR. These studies were further categorized into prediction of NOAF occurrence (n = 2) and detection of NOAF (n = 3) in ICU. The study design is summarized in the PRISMA flowchart reported in Fig. 1.

A comprehensive dataset comprising medical records from a total of 108,724 subjects was sourced from various databases, including Medical Information Mart for Intensive Care (MIMIC)–II [19], –III [20, 21], –IV [22], Kensington General Hospital (KGH) database [23], AmsterdamUMCdb [22], Ghent University Hospital database [22], and Belgian hospital ICU database [22]. The ICU admission records were retrieved for ML models design, testing, and external validation [22, 23]. The records of ICU admissions used in the modeling studies spanned the period from 2001 to 2020 [19–23]. The studies employed eight distinct ML models: K-nearest neighbor (K-NN) and support vector machine (SVM) were utilized in three studies [19–21], while forest (RF) and decision tree (DT) were applied in two studies [19, 21]. In addition, logistic reasoning [19], gradient boosted machine [19], CatBoost classification models [22], and deep learning models [23] were each analyzed in single studies.

The results of the selected studies are presented in two distinct sections: ML-based prediction of NOAF and ML detection of NOAF occurrence.

### AI-based prediction of NOAF occurrence

The prediction of NOAF occurrence in ICU patients was examined in two retrospective studies [19, 22]. These studies employed database analysis and reported models using the MIMIC-III [16] MIMIC-IV [22], AmsterdamUMC [22], and GUH [22] databases. The patients’ bedside records were collected between 2001 and 2012 [19], 2003 and 2016 [22], 2008–2019 [22] and 2013–2020 [22]. A comprehensive investigation encompassing a total of 107,463 cases is summarized in Table 2. In all the studies, ML-based models were explored, including K-NN, SVM, RF, LR, GBM, and DT [19], with one study incorporating CatBoost classification models [22]. Notably, one of the studies applied ML-based electrocardiogram (ECG) waveform processing techniques [22], while the second study predicted NOAF occurrence based on risk parameters such as age, chronic obstructive pulmonary disease, eGFR of 15 ml/min per 1.73 m^2^ or dialysis, emergency status, preoperative intra-aortic balloon pump need, valve surgery, and left ventricular ejection fraction < 30% [19].

**Table 2:** Prediction of occurrence of NOAF

Study (first author, year, ref)	ML method	Number of subjects	Performance of ML algorithms
J. Verhaeghe et al. 2023 [22]	CatBoost classification models	101,114	AUC = 0.81
			ECE = 0.04
			ESCE = 0.04
R. Karri et al. 2021 [19]	RF, DT, LR, K-NN, SVM, GBM	6349	GMB: AUC = 0.74 (0.71–0.77) LR: AUC = 0.73 (0.71–0.75) RF: AUC = 0.72 (0.69–0.75) K-NN: AUC = 0.68 (0.67–0.69) SVN: AUC = 0.67 (0.66–0.68) DT: AUC = 0.59 (0.55–0.63) POAF SCORE = 0.63 (0.62–0.64)
			GMB model had the highest sensitivity (0.74), DT had the highest specificity (0.84)
			All ML models outlined in this investigation, except for DT, outperformed the gold standard clinical scoring tool (POAF score)

Table reports the performance of ML algorithms achieved in each study in the prediction of occurrence of NOAF

*RF* random forest, *DT* decision tree, *LR* logistic regression, *K-NN* k-nearest neighbors, *SVM* support vector machine, *GBM* gradient boosted machine, *AUC* area under the curve

Each study evaluated the performance of their models using the area under the curve (AUC) (Table 2), achieving the best results of AUC = 0.81 [22] and AUC = 0.74 [19]. The authors of both publications assert the superiority of their models compared to other currently proposed ML-based AF prediction models [19, 22]. In addition, they claim superiority over a current gold standard clinical scoring tool, namely, POAF scoring system, which exhibited an AUC of 0.63 [19].

### ML-based detection of NOAF

The detection of NOAF in ICU patients was investigated in three retrospective studies [20, 21, 23] based on database analysis report models: MIMIC-III [20, 21]. The medical records were collected between 2001–2012 [20] and 2015–2020 [23]. A total of 1261 cases were investigated (Table 3). The three studies investigated ML-based models: K-NN [20, 21], SVM [20, 21], RF [20], and DA [21]. One study [23] did not explicitly specify the type of ML-based method utilized. Notably, all studies incorporated ML-based electrocardiogram (ECG) waveform processing techniques [20, 21, 23]. The performance evaluation in each study was conducted using various metrics. The best values, as presented in Table 3, were achieved by the study using F1 score (0.64–0.67) and expected calibration error (0.05–0.07) [23]. In another study, the SVM classifier demonstrated sensitivity greater than 98% and specificity exceeding 93% [20]. The authors of the three publications claim that their models are superior compared to other currently proposed ML-based AF detection models pointing out at a better sensitivity [23] and accuracy [20, 21].

**Table 3:** Detection of NOAF

Study (first author, year, ref)	ML method	Number of subjects	Performance of ML algorithms
B. Chen et al. 2022 [23]	Deep learning models	1043	Classification performance: F1 score (0.64–0.67), Calibration: expected calibration error (0.05–0.07)
S. Bashar et al. 2021 [20]	K-NN, SVM, RF	20	K-NN classifier: sensitivity = 84.01%, specificity = 64.55%, accuracy = 76.16%, PPV = 78.01% and NPV = 74.16%,
			SVM classifier: sensitivity = 98.18%, specificity = 93.98%, accuracy = 96.48%
			RF classifier: sensitivity = 97.78%, specificity = 90.38%, accuracy = 97.09%
S. Bashar et al. 2020 [21]	SVM, DT, k-NN	198	Overall confusion matrix: sensitivity = 100%, specificity = 98%, accuracy = 98,99%, PPV = 98%, NPV = 100%

Table reports the performance of ML algorithms achieved in each study in the detection of NOAF

*RF* random forest, *DT* decision tree, *LR* logistic regression, *K-NN* k-nearest neighbors, *SVM* support vector machine, *GBM* gradient boosted machine, *AUC* area under the curve, *ECE* expected calibration error, *ESCE* expected signed calibration error

## Discussion

This SR originally reports the available evidence on the role of ML-based prediction and detection models in patients with NOAF treated in ICU.

The first study compares ML-based predictive models capable of anticipating NOAF based on an extensive set of 194 variables including administered drug therapy, laboratory values, anthropometric data, and hemodynamic parameters. Notably, the designed models demonstrate the ability to predict NOAF onset 24–36 h before its occurrence [22]. The second study presents ML-based models that rely their prediction on demographics, physiological parameters, laboratory results, and clinical outcomes [19]. Although the specific timeframe for prediction is not explicitly specified in this article, the study remains valuable in the ICU clinical practice as it provides crucial information to intensivists regarding patients at risk of developing complications, such as NOAF. Based on the comparison of the performance of each ML-model measured in terms of AUC, it is observed that all ML models outperform traditional clinical scores and among the studied algorithms, CatBoost shows to be superior in comparison to the others. A noteworthy observation from the comparison of the ML models’ performance, measured in terms of the area under the curve (AUC), indicates that all ML models surpass traditional clinical scores. Furthermore, among the studied algorithms, CatBoost emerges as superior in comparison to the others. The selected articles suggest the potential capability of the described ML models to predict and detect NOAF, signaling a promising potential for a possible implementation of ML in both research and clinical practice. Importantly, ML methods exhibit an advantage over conventional clinical scoring systems, particularly in predicting the occurrence of NOAF and significantly limits human error and shortens detection process in unclear ECG readings. Moreover, ML methodologies significantly mitigate human error and expedite the detection process, especially in cases of unclear ECG readings.

Numerous research findings, comprehensive meta-analyses, and systematic reviews, consistently affirm the substantial potential in the utilization of ML methods. A study conducted in non-ICU setting stands as a pertinent example of leveraging ML for predicting NOAF [24]. Reliable outcomes have consistently demonstrated the effectiveness of ML predicting models, particularly in their ability to anticipate intensive care delirium up to 12 h in advance [25]. In addition, the use of ML has shown promise in predicting cardiovascular complications in diabetic patients [26].

Time factor is crucial in diagnosing NOAF in critical patients, given the elevated risk of death and the potential long-term complications [1–7]. The prediction and rapid detection of NOAF have been highly discussed in the medical society. The medical community has extensively discussed the importance of predicting and rapidly detecting NOAF. Numerous alternative strategies have been proposed for NOAF prediction, including assessing DNA methylation levels [27], measuring plasma aldosterone concentrations [28], and exploring immune-associated biomarkers [29]. While these methods indicate potential for NOAF prediction, further investigations through prospective clinical trials are essential. In this context, rapidly developing ML-based methods and their increasing integration into medical practice demonstrate a remarkable potential for the prediction and detection of NOAF. The agility and adaptability of ML approaches offer a promising avenue for timely identification and management of NOAF, potentially improving patient outcomes in critical care settings. Therefore, the evolving ML-based methods and their growing integration into medical practice hold substantial potential for the prediction and detection of NOAF.

Limitations of this SR include the relatively limited number of available studies, all of which were retrospective and based on few databases which could result in a partially overlapping of records. To address this, future prospective randomized clinical trials comparing the performance of ML-based models versus traditional clinical methods for the prediction and detection of NOAF in ICU are necessary. Such trials should provide more robust evidence and contribute to the refinement of ML applications in critical care settings.

## Conclusion

This SR provides a comprehensive summary of all available evidence related to the prediction and detection of NOAF in the ICU. Importantly, the evidence suggests that ML-based methods have already surpassed the POAF score, which currently serves as the gold standard in clinical practice. Among the various ML algorithms studied, CatBoost emerges as the top performer. Furthermore, the rapid development of ML signifies the potential for a paradigm shift, advocating for the redesign or integration of ML-based methods either independently or in conjunction with traditional risk scoring systems in the ICU. This recommendation is underscored by the need for further evaluation in prospective, randomized controlled trials, which will provide more robust insights into the efficacy and feasibility of ML applications in the critical care setting.

## Supplementary Information

Below is the link to the electronic supplementary material.

Supplementary file1 (PDF 93 kb)

## Data Availability

All data associated with this manuscript are included in the main text and Supplementary Materials.

## Abbreviations

AF: Atrial fibrillation
NOAF: New-onset atrial fibrillation
ICU: Intensive care unit
ML: Machine learning
RF: Random forest
DT: Decision tree
LR: Logistic regression
K-NN: K-nearest neighbors
SVM: Support vector machine
GBM: Gradient boosted machine
AUC: Area under the curve
ECE: Expected calibration error
ESCE: Expected signed calibration error
PRIMSA: Preferred Reporting and Items for Systematic Reviews and Meta-Analyses
SR: Systematic review
