# Supplementary material for: Machine learning in the prediction and detection of new-onset atrial fibrillation in ICU: a systematic review
Source: J Anesth. 2024 Apr 9;38(3):301–8. doi: 10.1007/s00540-024-03316-6 (PMC11096200; doi:10.1007/s00540-024-03316-6)
Supplement: Supplementary file 1 — Supplementary file1 (PDF 93 kb) [file 540_2024_3316_MOESM1_ESM.pdf]

Search Name: **PUBMED**
Date Run: 3/07/2023

Search: **(atrial fibrillation OR new onset) AND (Artificial intelligence OR machine learning OR deep learning or neural network) AND (intensive care OR critical patients OR ICU)** Sort by: **Publication Date**
("atrial fibrillation"[MeSH Terms] OR ("atrial"[All Fields] AND "fibrillation"[All Fields]) OR "atrial fibrillation"[All Fields] OR ("new"[All Fields] AND ("age of onset"[MeSH Terms] OR ("age"[All Fields] AND "onset"[All Fields]) OR "age of onset"[All Fields] OR "onset"[All Fields] OR "onsets"[All Fields] OR "onsetting"[All Fields]))) AND ("artificial intelligence"[MeSH Terms] OR ("artificial"[All Fields] AND "intelligence"[All Fields]) OR "artificial intelligence"[All Fields] OR ("machine learning"[MeSH Terms] OR ("machine"[All Fields] AND "learning"[All Fields]) OR "machine learning"[All Fields]) OR ("deep learning"[MeSH Terms] OR ("deep"[All Fields] AND "learning"[All Fields]) OR "deep learning"[All Fields]) OR ("neural networks, computer"[MeSH Terms] OR ("neural"[All Fields] AND "networks"[All Fields] AND "computer"[All Fields]) OR "computer neural networks"[All Fields] OR ("neural"[All Fields] AND "network"[All Fields]) OR "neural network"[All Fields])) AND ("critical care"[MeSH Terms] OR ("critical"[All Fields] AND "care"[All Fields]) OR "critical care"[All Fields] OR ("intensive"[All Fields] AND "care"[All Fields]) OR "intensive care"[All Fields] OR (("critical"[All Fields] OR "critically"[All Fields]) AND ("patient s"[All Fields] OR "patients"[MeSH Terms] OR "patients"[All Fields] OR "patient"[All Fields] OR "patients s"[All Fields])) OR ("intensive care units"[MeSH Terms] OR ("intensive"[All Fields] AND "care"[All Fields] AND "units"[All Fields]) OR "intensive care units"[All Fields] OR "icu"[All Fields]))

Total 200

Search Name: **Scopus**
Date Run: 12/07/2023

Search: **(atrial fibrillation OR new onset) AND (Artificial intelligence OR machine learning OR deep learning or neural network) AND (intensive care OR critical patients OR ICU)**

Total 298

Search Name: **MEDLINE**
Date Run: 18/07/2023

Search: **(new onset atrial fibrillation) AND (Artificial intelligence OR machine learning OR deep learning) AND (intensive care unit OR ICU**

(new[All Fields] AND ("age of onset"[MeSH Terms] OR ("age"[All Fields] AND "onset"[All Fields]) OR "age of onset"[All Fields] OR "onset"[All Fields]) AND ("atrial fibrillation"[MeSH Terms] OR ("atrial"[All Fields] AND "fibrillation"[All Fields]) OR "atrial fibrillation"[All Fields])) AND (("artificial intelligence"[MeSH Terms] OR ("artificial"[All Fields] AND "intelligence"[All Fields]) OR "artificial intelligence"[All Fields]) OR ("machine learning"[MeSH Terms] OR ("machine"[All Fields] AND "learning"[All Fields]) OR "machine learning"[All Fields]) OR ("deep learning"[MeSH Terms] OR ("deep"[All Fields] AND "learning"[All Fields]) OR "deep learning"[All Fields])) AND (("intensive care units"[MeSH Terms] OR ("intensive"[All Fields] AND "care"[All Fields] AND "units"[All Fields]) OR "intensive care units"[All Fields] OR ("intensive"[All Fields] AND "care"[All Fields] AND "unit"[All Fields]) OR "intensive care unit"[All Fields]) OR ("intensive care units"[MeSH Terms] OR ("intensive"[All Fields] AND "care"[All Fields] AND "units"[All Fields]) OR "intensive care units"[All Fields] OR "icu"[All Fields])) AND (medline[sb] AND "2019/01/16"[PDat] : "2023/07/18"[PDat])

Total 719

Search Name: **EMBASE**
Date Run: 23/07/2023

Search: **(new onset atrial fibrillation) AND (Artificial intelligence OR machine learning OR deep learning) AND (intensive care unit OR ICU**

(new[All Fields] AND ("age of onset"[MeSH Terms] OR ("age"[All Fields] AND "onset"[All Fields]) OR "age of onset"[All Fields] OR "onset"[All Fields]) AND ("atrial fibrillation"[MeSH Terms] OR ("atrial"[All Fields] AND "fibrillation"[All Fields]) OR "atrial fibrillation"[All Fields])) AND (("artificial intelligence"[MeSH Terms] OR ("artificial"[All Fields] AND "intelligence"[All Fields]) OR "artificial intelligence"[All Fields]) OR ("machine learning"[MeSH Terms] OR ("machine"[All Fields] AND "learning"[All Fields]) OR "machine learning"[All Fields]) OR ("deep learning"[MeSH Terms] OR ("deep"[All Fields] AND "learning"[All Fields]) OR "deep learning"[All Fields])) AND (("intensive care units"[MeSH Terms] OR ("intensive"[All Fields] AND "care"[All Fields] AND "units"[All Fields]) OR "intensive care units"[All Fields] OR ("intensive"[All Fields] AND "care"[All Fields] AND "unit"[All Fields]) OR "intensive care unit"[All Fields]) OR ("intensive care units"[MeSH Terms] OR ("intensive"[All Fields] AND "care"[All Fields] AND "units"[All Fields]) OR "intensive care units"[All Fields] OR "icu"[All Fields])) AND (embase[sb] AND "2019/01/16"[PDat] : "2023/07/23"[PDat])

Total 379
